# Modelling Diabetic Cardiomyopathy: Using Human Stem Cell-Derived Cardiomyocytes to Complement Animal Models

**DOI:** 10.3390/metabo12090832

**Published:** 2022-09-03

**Authors:** Ujang Purnama, Marcos Castro-Guarda, Om Saswat Sahoo, Carolyn A. Carr

**Affiliations:** 1 Department of Physiology, Anatomy and Genetics, University of Oxford, Oxford OX1 3PT, UK; 2 Department of Biotechnology, National Institute of Technology Durgapur, Durgapur 713216, India

**Keywords:** hiPSCs, cardiomyocytes, 3D cardiac organoids, engineered heart tissue, maturation, in vitro disease modelling, animal models, metabolism, diabetic cardiomyopathy, diabetes, heart disease, environmental factors

## Abstract

Diabetes is a global epidemic, with cardiovascular disease being the leading cause of death in diabetic patients. There is a pressing need for an in vitro model to aid understanding of the mechanisms driving diabetic heart disease, and to provide an accurate, reliable tool for drug testing. Human induced-pluripotent stem cell-derived cardiomyocytes (hiPSC-CMs) have potential as a disease modelling tool. There are several factors that drive molecular changes inside cardiomyocytes contributing to diabetic cardiomyopathy, including hyperglycaemia, lipotoxicity and hyperinsulinemia. Here we discuss these factors and how they can be seen in animal models and utilised in cell culture to mimic the diabetic heart. The use of human iPSC-CMs will allow for a greater understanding of disease pathogenesis and open up new avenues for drug testing.

## 1. Introduction

Diabetes is a global epidemic problem, affecting nearly half a billion people worldwide and projected to increase by 51%, surpassing 578 million and 700 million individuals by 2030 and 2045, respectively [1]. The development of diabetes increases the risk of cardiovascular disease, such that 30% of diabetic patients are diagnosed with cardiovascular disease, and this is the leading cause of mortality for individuals with diabetes [2]. Diabetes not only increases vascular disease but also affects the cardiomyocytes themselves, as shown by Rubler and colleagues 50 years ago when they coined the term diabetic cardiomyopathy (DbCM). Numerous studies have since been performed, both in animal models and in diabetic patients, yet the underlying pathophysiological mechanisms of DbCM remain poorly understood. Studies of patients are predominantly limited to non-invasive techniques due to the difficulty in obtaining human heart tissue. Samples primarily come from biopsies of end-stage heart failure patients and therefore cannot model the early stages of diabetes and are often affected by other co-morbidities.

Therefore, it is essential to develop a robust in vitro model that can elucidate the key mechanisms driving DbCM and provide an accurate, predictive tool for drug testing. Primary human cardiomyocytes are difficult to obtain, limited in numbers and cannot be maintained in culture for more than a few days. Similarly, adult rodent cardiomyocytes have a short lifetime in culture, and neonatal rodent cardiomyocytes do not fully represent the adult human heart. There are growing concerns that rodent cardiomyocytes and animal models do not recapitulate the human disease sufficiently, as many therapeutic compounds have shown promise during pre-clinical testing but do not perform as predicted in patients. Human induced pluripotent stem cell-derived cardiomyocytes (hiPSC-CM) may provide a viable alternative to complement animal models and can be obtained in unlimited numbers. In addition, hiPSC-CM can be genetically modified and have been used to model several inherited cardiac disorders in vitro [3,4,5].

## 2. Causes of Diabetes and Induction of Diabetic Cardiomyopathy (Insulin Resistance)

Diabetes mellitus (DM) is broadly classified into Type 1 diabetes mellitus (T1DM) and Type 2 diabetes mellitus (T2DM). T1DM, being a consequence of autoimmune β-cell destruction, results in hyperglycaemia, with a relative absence of insulin production. In contrast, T2DM results from both genetic and environmental factors, and is characterised by systemic insulin resistance, hyperglycaemia, hyperlipidaemia and abnormal secretion of insulin.

Within the heart, DM is characterised by structural and functional alterations, activation of inflammatory processes and metabolic dysregulation. A multitude of mechanisms have been reported in the pathogenesis of diabetic cardiomyopathy including alterations in cellular energy metabolism, increased lipid oxidation, intracellular accumulation of glycerides, mitochondrial dysfunction, abnormal calcium signalling, cardiomyocyte hypertrophy and apoptosis [6]. These trigger alterations in downstream transcription factors that lead to changes in gene expression, myocardial substrate utilisation, myocyte growth, endothelial function and myocardial compliance, so that diabetic cardiomyopathy is a complex and multifactorial disease. The clinical progression of DbCM is characterised by cardiac hypertrophy and diastolic dysfunction. This may increase the risk of pulmonary congestion and can progress to heart failure with preserved ejection fraction (HFpEF). If additional stressors are present, such as hypertension or ischemia, patients may go on to develop systolic dysfunction [7]. T1DM results in loss of cardiomyocytes and increased collagen deposition and impaired systolic function tends to occur earlier in these patients [8].

## 3. Molecular and Metabolic Alterations of Diabetic Cardiomyopathy

Multiple mechanisms are involved in the progression of DbCM, which we discuss in turn in the next section. An understanding of the factors that drive changes within the myocardium in diabetes is important for understanding disease pathogenesis, and what is required in an in vitro model, and for identifying new targets for therapy.

### 3.1. Changes in Substrate Metabolism

**Figure 1:**
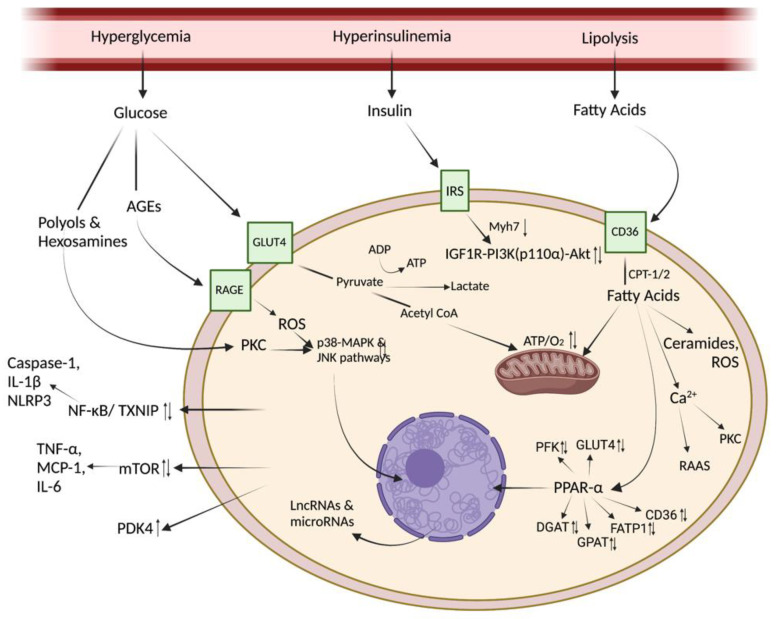
Molecular mechanisms inducing diabetic cardiomyopathy. Elevated levels of plasma glucose and insulin in T2DM and increased lipolysis of fatty acids activates a number of adverse signaling pathways in cardiomyocytes. (Image created with BioRender.com).

The diabetic heart is bathed in an abnormal composition of substrates, due to the hyperglycaemia, hyperlipidaemia and circulating cytokines, which changes the fuel choice of the heart and has subsequent effects on intracellular processes. The healthy heart obtains its energy from a combination of fatty acids, glucose, amino acids and ketone bodies, and has the flexibility to shift between substrates according to the underlying conditions. In the diabetic heart, metabolic preference changes, with the heart metabolising predominantly fatty acids, with glucose metabolism only contributing a small amount to overall energy generation. The flexibility of substrate metabolism is lost, in part due to the high plasma fatty acid concentrations in the circulation [9]. Increased expression and localisation of the fatty acid transporter FAT/CD36 to the plasma membrane of cardiomyocytes result in increased cardiac fatty acid uptake, which fuels increased mitochondrial fatty acid oxidation and myocardial triglyceride esterification in diabetes. Increased fatty acid metabolism drives decreased metabolism of alternative substrates which, in combination with myocardial insulin resistance, decreases glucose uptake, glycolysis and glucose oxidation within the diabetic heart. Moreover, the use of ketone bodies and certain amino acids and their derivatives showed enhanced metabolism in diabetic cardiomyocytes in both in vivo and in vitro diabetic models [10]. This metabolic dysfunction in diabetes has consequences beyond our traditional view of metabolism, as changes in intracellular metabolites results not only in changes in energy production within the heart, but also has consequences for metabolite-sensitive signalling pathways that regulate cell structure, function and survival as shown in Figure 1.

### 3.2. Glucotoxicity

Hyperglycaemia occurs as a result of decreased glucose clearance and increased hepatic gluconeogenesis. Chronic exposure to hyperglycaemia can result in cellular impairment and may become irreversible over time through a process called glucotoxity [11]. Several mechanisms play a role in DbCM as a result of hyperglycaemia. The three main mechanisms are the elevation of advanced glycation end products (AGEs), the increase in hexosamine and polyol flux and the activation of protein kinase C (PKC), shown in Figure 1 and summarised in Table 1 [12]. AGEs are produced through a non-enzymatic reaction between protein and lipids in the presence of reducing sugars and are found at high levels in the plasma of diabetic patients. They adversely affect cellular processes by directly trapping and cross-linking proteins or by binding to receptors for signalling pathways [13,14]. Advanced glycation end-product generation leads to the formation of reactive oxygen species (ROS), oxidative stress and altered gene expression. Furthermore, increased polyol pathway flux leads to inhibition of glyceraldehyde-3-phosphate dehydrogenase (GAPDH), which modifies the pathway of glycolysis and eventually leads to glucotoxicity [15]. Finally, PKC can lead to activation of the pathways involved in p38 mitogen-activated protein kinase (MAPK) and c-Jun N-terminal kinase (JNK), which contribute to cardiac hypertrophy [16].

### 3.3. Lipotoxicity

Excessive esterification of fatty acids into triglycerides and ceramides within the diabetic myocardium has been shown to stimulate oxidative stress, mitochondrial damage and cardiomyocyte apoptosis [24,25]. Collectively, these studies suggested that increases in fatty acid supply in the presence of diabetes alter cardiac lipid homeostasis and result in lipotoxicity as seen in Figure 1. Excessive fatty acid oxidation leads to enhanced PGC1α/PPARα activation, which drives fatty acid-mediated transcriptional regulation within the heart, contributing to cardiac hypertrophy and hypoxia. Sustained increase in the secretion of cytokines and chemokines such as TNF-α, MCP-1, IL-6, as a consequence of increased fatty acid oxidation, also exerts autocrine/paracrine effects on cardiomyocytes leading to injuries and heart failure.

Palmitic and oleic acid account for more than half of the total plasma fatty acids released by adipose tissues into the bloodstream [26,27]. However, long-chain saturated fatty acids, such as palmitate, exhibit several cytotoxic effects which are not seen with unsaturated fatty acids such as oleate. Dyntar et al. showed that palmitic acid induces apoptosis in adult rat cardiomyocytes via de novo formation of ceramides and activation of the mitochondrial apoptosis pathway [28]. Ceramide synthesis also inhibits mitochondrial β-oxidation of fatty acids which activates the NLRP3 inflammasome and initiates inflammatory mechanisms linked to NF-ĸB mediated IL-6 and TNF-α [21,22]. Similarly, Oh et al. showed that high concentrations of saturated fatty acids triggered the p38α/MAPK pathway and mediated cardiomyocyte apoptosis. They further postulated that palmitic acids upregulated c-fos, c-jun and caspase-8 leading to cell death [23]. More recently palmitic acids have been postulated to induce ferroptosis, triggered by the accumulation of iron and lipid hydroperoxides [29]. Enhanced palmitic acid levels also induce microRNAs such as miR-451, which suppress the LKB1/AMPK pathway and lead to cardiac hypertrophy [30] and drive mitochondrial ROS generation, leading to endoplasmic reticulum stress and apoptosis of cardiomyocytes [31]. In contrast, oleic acid has been shown to be beneficial to cardiovascular cells [22]. Pretreatment with oleate has been seen to nullify the activation by palmitate of the TNF-α mediated JNK1/2 pathway and NF-ĸB mediated pathways, thus providing an anti-inflammatory environment [22].

### 3.4. Oxidative Stress and Mitochondrial Coupling

The metabolic changes induced by diabetes generate a redox imbalance in the cardiomyocytes which culminates in elevated ROS production and oxidative stress. Hyperglycaemia induces excessive superoxide production in mitochondria which have been shown to be more susceptible to mitochondrial permeability transition pore (MPTP) opening in some, but not all, rodent models of diabetes [32,33]. Moreover, it has been described that hyperglycaemia elevated levels of protein glycosylation and dyslipidemia, which upregulated NADPH oxidase activities and dramatically increased the ROS content in DbCM [34,35]. Finally, as a consequence of the increased use of NADPH to produce sorbitol by the reduction of glucose (increase polyol pathway flux), the NADPH/NADP+ ratio declines, which depletes NAPDH availability to maintain glutathione in its reduced form [36], required for defence against ROS.

The increased beta-oxidation rate in the mitochondria increases the delivery of electron donors (NADH and FADH_2_) to the electron transport chain. The excess of these electrons interacts with molecular oxygen and culminates in superoxide formation, followed by ROS overproduction within the mitochondria [37,38,39], which finally drives mitochondrial and cardiac dysfunction [40,41]. The increased fatty acid oxidation and ROS generation within the diabetic heart upregulate the expression and activity of the mitochondria uncoupling proteins (UCP), namely UCP2 and UCP3 [42,43]. These proteins disrupt the proton gradient in the mitochondria, generating an increase in oxygen consumption which is accompanied by a reduction in the ATP/oxygen ratio, resulting in a decrease in cardiac efficiency [42,44,45]. Both oxidative stress and UCPs have been implicated in the development of diabetic complications at the cardiovascular level [46,47].

### 3.5. Impaired Ion Channels Function and Calcium Homeostasis

T2DM patients and STZ-induced T2DM models show a series of deviations in the electrocardiogram. Characteristics such as prolonged QT interval, atrial fibrillation, increased arrhythmogenic potential and an enhanced disparity in depolarisation-repolarisation were noticed in such models. Elevation in plasma glucose concentration has been associated with significant changes in particular sodium and potassium ion channels. For example, an uneven decrease of I_K_ dysregulated several repolarisation procedures, thereby increasing the action potential duration and explaining the episodes of ventricular arrhythmia is seen in some diabetic patients [48]. This increase in action potential duration, consistent with reduced expressions of Kcnd2, Kcnd3 and Kcnip2 (genes encoding for K^+^ channel alpha-subunits), has also been associated with atrial fibrillation in diabetic db/db mice [49]. In diabetic cardiomyocytes, reduced Na^+^ influx during contractions has been associated with a reduction in the sodium current I_Na_ and an increased intracellular calcium concentration [50]. Depressions in repolarising K^+^ currents due to hyperglycaemia showed prolonged and increased temporal intervals of the repolarisation phase in myocytes, thus remodelling the electrical phenotype of the diabetic heart [51]. Myocyte alterations were also observed due to downregulation of HCN2 and HCN4, thus projecting an altered arrhythmogenic profile of the diabetic heart [52].

Impaired cytosolic and mitochondrial Ca^2+^ handling has been detected in rodent models of both T1DM and T2DM [53]. In the cytosol, decreased expression of sarcoplasmic/endoplasmic reticulum calcium ATPase 2 (SERCA 2a) in diabetic hearts was shown to result in decreased contractile function [54]. High glucose levels have been shown to impair mitochondrial Ca^2+^ uptake and reduce mitochondrial Ca^2+^ content [Ca^2+^]_m_ [32,55]. The activity of complexes in the electron transport chain are enhanced by increased [Ca^2+^]_m_, as is the transport of glucose through the pyruvate dehydrogenase complex into the Krebs cycle, so that a decrease in [Ca^2+^]_m_ directly impacts the mitochondrial energy generation [55].

Impairment of Ca^2+^ handling, activation of RAAS (renin-angiotensin-aldosterone system), ER stress and other pathophysiological aberrations have been seen to promote the activation of protein kinase C (PKC). Isoforms of PKC (PKC α1 and PKC β2) have been seen to be involved in the development of cardiac hypertrophy. They induce a series of changes in cellular functions and enhance extracellular matrix, hypertrophy and apoptosis in DbCMs, which ultimately leads to heart failure [56].

## 4. Modelling the Disease

Animal models are a versatile research tool to increase our understanding of the mechanisms implicated in diabetic cardiomyopathy and are particularly valuable in exploring the complex interactions of insulin signalling on different organs of the body. Researchers have been modelling DbCM using large and small animal models for many years. Due to ease of maintenance, short breeding cycles and the high homology of the human and mouse genome, the latter has been widely used.

Animal models can be broadly divided into three groups: genetic models, chemically induced or diet-induced models. T1D can be induced using high concentrations of streptozotocin (STZ), which kills the pancreatic beta cells and results in a model of uncompensated T1D [57]. Genetic models of T1D include the NOD mouse, an inbred non-obese diabetic model, and the Akita mouse which has a genetic mutation leading to progressive loss of beta cells after birth [57]. T2D is studied in the ob/ob and db/db mice and the ZDF rat, which have mutations in leptin and leptin receptor genes [58]. However, there are concerns those cardiac effects may not result from the hyperphagia and obesity but instead from the changes in leptin signalling itself. Nevertheless, these animals are obese and have elevated plasma glucose and fatty acids and therefore do recapitulate many aspects of the T2D phenotype. Alternatively, the Goto Kakizaki (GK) rat is a non-obese T2DM animal model produced by inbreeding of Wistar rats with elevated plasma glucose levels [59]. This model has been widely used to study the effect of glucotoxicity on cardiac function, such as the relationship between T2DM with mitochondrial oxidative stress [60] or LV remodelling [61]. T2D can also be induced using high fat or high fructose diets. Some mouse strains, such as the C57BL/6J mouse, are prone to insulin resistance when fed a high-fat diet [62]. Alternatively, a combination of high-fat feeding and a low dose STZ injection can induce a clinically relevant model of the early stages of T2D [63]. The high fructose diet model results in hyperinsulinemia and more pronounced glucose intolerance [64] resulting in insulin resistance and cardiac pathology [65].

Although these models give valuable insights into the causes of DbCM, translation to the human cardiomyocyte is also needed. The rodent heart, although being similar to the human heart in structure [62], differs from it both genetically and physiologically [66]. In contrast to the average human heart rate of 50–60 bpm, the rat heart beats at around 400 bpm and the mouse heart at around 500 bpm. The ion channels used to drive contraction differ between rodents and humans, as does the expression of alpha and beta myosin heavy chains between the ventricles [67]. More subtle differences in protein expression can result in significant differences in physiology. For example, the mouse model for Duchenne muscular dystrophy (DMD), the Mdx mouse, has a normal life span and develops cardiac dysfunction at a much later timepoint than seen in DMD patients. Although the Mdx mouse lacks dystrophin, it has upregulated expression of utrophin to compensate, which is not seen in DMD patents, and which ameliorates the dystrophic cardiomyopathy [68]. Human cell models are therefore needed to confirm that changes in gene and protein expression seen in rodent models can also be identified within a human heart cell.

## 5. In Vitro Models of Insulin-Resistant Cardiomyocytes

Various in vitro models of insulin-resistant cardiomyocytes have been developed using rodent neonatal cardiomyocytes, H9C2 rat cardiovascular myoblasts or the HL-1 immortalised mouse cardiomyocyte line cultured with high palmitate levels [69] as shown in Table 2 and Table S1. However, the cell lines have lower expression and disorganisation of contractile proteins compared to adult cardiomyocytes and comparative transcriptomic analysis showed distinct differences in phenotype and gene expression patterns in these cells [70]. Primary cardiomyocytes from adult rat and mouse heart do not survive for long period of time in culture which limits their usage. Neonatal rat and mouse cardiomyocytes can be cultured for longer but maintain a neonatal phenotype [70]. Furthermore, although useful in vitro models to validate changes seen in vivo in the rodent heart, these cells do not provide translation to the human heart.

The immortalised human cardiomyocyte line, AC16, has been used to study heart disease. However, in terms of metabolism, hypoxic sensitivity and gene expression profiling, these cells differ from adult cardiomyocytes [70]. More recently, human induced-pluripotent stem cell-derived cardiomyocytes (hiPSC-CM) have shown the potential to provide a bridge to translation. There have been numerous reviews about the benefit of iPSC-CM to become a versatile tool for disease modelling and drug targeting [71]. However, unlike other monogenic diseases that have been successfully studied in vitro using human iPSC-CM, DbCM is a multifactorial and lifestyle-related disease, and therefore the challenges of using human iPSC-CM to model the diabetic heart are far greater.

### 5.1. Human Induced Pluripotent Stem Cell-Derived Cardiomyocytes (hiPSC-CM)

To successfully model the adult human heart and provide a suitable model to translate results seen in rodent models, it is necessary to confirm that hiPSC-CM recapitulate the structure and metabolism of the mature cardiomyocyte. When first differentiated, hiPSC-CM retain an immature phenotype and differ from adult cardiomyocytes in gene expression [72,73], structural features [74], metabolism [75], calcium handling [76] and contractile function [77]. With particular relevance to modelling the diabetic heart, there are differences in substrate utilisation between neonatal and adult cardiomyocytes. In the neonatal heart, glycolysis remains the primary source of cellular energy, whereas fatty acid oxidation provides about 80% of the energy for mature cardiomyocytes. Other substrates such as glucose, which are regulated by glucose transporters GLUT1 and GLUT4, serve as additional fuels for nutrition [6]. Activation of protein kinase B/Akt signalling pathway via phatidylinositol-3-kinase (PI3K) mediates glucose uptake and FAT/CD36 translocation, enhancing the uptake of fatty acids. Upregulation of peroxisome proliferator-activated receptor α (PPARα) and PPAR-γ co-activator-1α (PGC-1α) enhance fatty acid β oxidation and mitochondrial biogenesis [6]. With maturation, mitochondria in adult cardiomyocytes also undergo morphological changes with alignment with myofibrils and increased mitochondrial density to enhance their oxidative capacity [76,78]. When first differentiated, hiPSC-CM have been shown to have low expression of GLUT4 and therefore they did not exhibit insulin-stimulated glucose uptake [79].

There are many approaches to induce the maturation of hiPSC-CM through biochemical factors, genetic manipulation, co-culture, electrical stimulation, substrate stiffness, mechanical stretch and 3D culture which are discussed in detail by Karbassi et al. [76]. These strategies are often combined to increase the maturation of the hiPSC-CM. We and others have shown that addition of fatty acids to cell culture media induces structural and metabolic maturation of differentiated hiPSC-CM [80] and that culture of hiPSC-CM in 3D best mimics the physiological properties of adult cardiomyocytes [81,82,83,84,85,86]. The 3D culture models fall into two categories, engineering the cells using a scaffold (typically hydrogel) between two silicon posts as engineered heart tissue (EHT) [83,87,88,89] and self-assembly formation in 3D organoids without the addition of extracellular matrix/scaffolds [90,91,92,93]. Murine/hiPSC-derived 3D cardiac models, including spherical aggregates of cardiomyocytes, engineered heart tissues, cardiac organoids, vascularised microchambers etc., have been established to recapitulate cardiac-specific organisation in vitro [92,93,94,95,96,97,98,99].

The advancement of human 3D cardiac organoids allows us to model complex diseases caused by genetic mutation and environmental factors. The human 3D organoids created by co-culturing different type of cells such as cardiomyocytes, fibroblasts and endothelial cells have been shown to recapitulate the differences between cardiomyocytes from genetic cardiomyopathies versus healthy cardiomyocytes [91]. Furthermore, the complex structure of 3D cardiac organoids allows us to look at the oxygen diffusion as a gradient which better recapitulates the hallmarks of the acute post-myocardial infarction state [92]. It should be remembered, however, that 3D models are limited by the inability to model the structural and functional complexity of the tissues and organs they intend to represent [100,101,102,103,104,105]. Nevertheless, these cardiac organoids can become fruitful models to study diabetic cardiomyopathy.

### 5.2. Two-Dimensional (2D) and 3D hiPSC-CM for Modelling Diabetic Cardiomyopathy

Previous studies have suggested that to model DbCM using hiPSC-CM, the cells should meet the minimum requirement of DbCM etiology and show insulin resistance, a metabolic shift, lipotoxicity, hypertrophy and altered cardiac function [106]. These changes can be induced by culturing the cells in the extracellular milieu found in the diabetic state. Drawnel et al. used media containing fatty acids in the absence of glucose to induce a metabolic shift toward more fatty acid oxidation. The cells were then cultured in media consisting of intermediate glucose levels, endothelin 1 and cortisol. Subsequently, the cells started showing hallmarks of their counterparts in the diabetic heart, with glucose-restriction-induced cellular distortion, lipid accumulation and peroxidation, decreased frequency of calcium transients, cellular hypertrophy and loss of sarcomere integrity [107]. Graneli et al. used a mixture of linoleic and oleic acids to mature the hiPSC-CM and induced insulin resistance by 4 days of culture with fatty acids but no glucose, followed by 6 days with high levels of glucose and palmitate supplemented with uric acid and endothelin-1 [106]. They showed an increase in basal respiration but a decrease in maximal respiration suggestive of mitochondrial dysfunction. Geraets et al. cultured embryonic stem cell-derived cardiomyocytes with high palmitate and insulin and saw lipid-induced insulin resistance [108], although they commented that the cells were structurally and functionally immature. The toxic effect of ceramide accumulation in diabetic cardiomyocytes was demonstrated by Bekhite et al. in hiPSC-CM treated with insulin, palmitic acid, glucose with endothelin-1 and cortisol [25]. They reported a decrease in beating rate and expression of SERCA2a, with increased mitochondrial ROS and oxidative damage. These effects were recapitulated by induction of endogenous ceramide in hiPSC-CM which induced mitochondrial fragmentation and elevated mitophagy.

Interestingly, Burkart et al. showed that mutations in insulin regulatory genes alter the mitochondrial size, DNA content and ROS production in undifferentiated hiPSC, resulting in impaired oxidative metabolism. The use of hiPSC from patients revealed that it was the insulin resistance that drove the impairment in energy homeostasis and increased oxidative stress [91]. Further work from this group showed that insulin-resistant skeletal myoblasts, differentiated from hiPSC from patents with a monogenic form of insulin resistance, showed impaired insulin signalling and aberrant glucose uptake and glycogen synthesis [109,110].

We have induced insulin resistance using a staged approach of culture for a week in maturation media containing 5 mM glucose supplemented with 0.4 mM oleic acid, followed by glucose-free insulin resistance media containing 0.3 mM palmitic acid and 50 nM insulin, and then a further three days with 12 mM glucose added to the insulin resistance media [111]. We showed that the insulin-resistant hiPSC-CM demonstrated an impaired hypoxic response previously identified in rodent cells [112] and that this could be rescued by treatment with the drug Molidustat which stabilizes the hypoxic response.

Lewis-Israeli et al. have developed the most complex model of the developing heart, using differentiating iPSCs, which contain multi-lineage cardiac cell types and internal chambers. They modelled the effects of pre-gestational diabetes by exposing a pre-prepared heart organoid to diabetic levels of glucose and insulin [113]. They saw structural and metabolic changes including cardiac hypertrophy, reduction in mitochondrial number and size, irregular frequency of action potentials, aberrant lipid metabolism and increased glycolysis, thus recapitulating several factors of diabetic cardiomyopathy. Advanced glycation end-products were used by Wang et al. to induce diabetic cardiomyopathy in 3D engineered cardiac tissues. Murine-based cardiac tissue models were treated with multiple dosages of AGEs, which led to the stimulation of several markers of fibrosis, inflammation and oxidative stress, thus serving as a robust 3D in vitro model to study the cellular and molecular features of diabetic cardiomyopathy [114].

## 6. Conclusions

Validating cardiomyocyte-specific effects in human cells is essential for drug development and progression to clinical trials. The recent advancement of human induced-pluripotent stem cells makes it possible to culture human cardiomyocytes in the lab for long periods of time. Using defined culture conditions to induce insulin resistance, iPSC-CM show great potential to study the effects of diabetes in cardiomyocytes which may lead to diabetic cardiomyopathy. The application of more complex cell models, such as engineered heart tissue and organoids, will generate even more relevant in vitro models to complement studies using animal models. This will advance our understanding of the more complex effects of diabetes and provide a testbed for drug development.

## Figures and Tables

**Table 1:** Metabolic changes leading to diabetic cardiomyopathy.

Triggers	Mediators and Mechanism of Action	Consequences
Hyperglycaemia (glucotoxicity)	Increased AGE [17], hexosamine flux [18], polyol flux [19], protein C kinase activation [20]	Decreased myocyte contractility, increased ventricular stiffness, prolonged calcium transients, impaired relaxation and increased myocytes apoptosis
Hyperlipidaemia (lipotoxicity)	Enhanced PGC1α/PPARα, increased secretion of cytokines (TNF-α, MCP-1, IL-6) [21,22], triggered p38α/MAPK pathway, upregulated c-fos, c-jun and caspase-8 [23]	Hypertrophy, heart injury, cardiomyocyte apoptosis, cell death

**Table 2:** Different in vitro cell models to study insulin resistance in cardiomyocytes.

**Most used in-vitro cell models**	**Benefits** Ease of maintenance Relatively large commercial availability Simple culturing protocol	**Limitations** Loss of specific cardiac features Resistance to hypoxic injury Highly glycolytic and use high glucose for energy generation
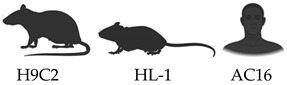		
**Potential future in-vitro models**	Myocardial gene expression more closely resembles that in human adult cardiomyocytes More responsive to hypoxic injury More mature cells show increased fatty acid oxidation	High degree of variability in some hiPSC-CM Require extra steps to create more mature cardiomyocytes
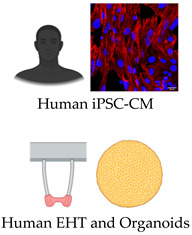		

H9C2: female embryonic BDIX rat ventricular tissue, HL-1: immortalised mouse atrial cardiomyocytes, AC16: immortalised human ventricular cardiomyocytes fused with transformed human fibroblast, hiPSC-CM: Human induced-pluripotent stem cells-derived cardiomyocytes, EHT: Engineered heart tissue.

## Supplementary Materials

The following supporting information can be downloaded at: https://www.mdpi.com/article/10.3390/metabo12090832/s1. Table S1: Different ways to model the effect of diabetes on the heart in vitro. References [21,106,107,111,112,115,116,117,118] are cited in the supplementary materials.
