# Supplementary material for: Modelling Diabetic Cardiomyopathy: Using Human Stem Cell-Derived Cardiomyocytes to Complement Animal Models
Source: Metabolites. 2022 Sep 3;12(9):832. doi: 10.3390/metabo12090832 (PMC9503602; doi:10.3390/metabo12090832)
Supplement: Supplementary file 1 [file metabolites-12-00832-s001.zip › metabolites-1832669-supplementary.pdf]

Supplementary

Table S1. Different Ways to Model the Effect of Diabetes on the Heart In Vitro

In vitro model	Glucose	Palmitic acid	Insulin	Other substrates	Duration
Human iPSC-CMs in 2D monolayer culture	10 mM	-	-	10 nM Endothelin-1 and 1 mM cortisol	2 days [112]
	12 mM	300 µM	50 nM	-	6 days [25]
	5.5 mM	500 µM	100 nM		24 hours [113]
	20 mM	50 µM		15 mg/dl uric acid and 10 nM Endothelin-1	6 days [105]
HL-1 cells in 2D monolayer culture	25 mM	20 µM	50 nM	-	16 hours [114]
	5.5 mM	500 µM	50 nM	-	24 hours [109]
	5.5 mM	500 µM	100 nM	-	24 hours [113]
H9C2 rat heart-derived embryonic myocytes	5.5 mM	-	-	15 mg/dl uric acid	24 hours [107]
Primary neonatal rat cardiomyocytes in 2D monolayer culture	30 mM	300 µM	-	-	24 hours [115]
	5.5 mM	-	-	15 mg/dl uric acid	24 hours [107]
Primary neonatal rat heart cells in EHT culture	25 mM	-	-	-	8 days [116]
